# Cytochrome P450s in human immune cells regulate IL-22 and c-Kit via an AHR feedback loop

**DOI:** 10.1038/srep44005

**Published:** 2017-03-09

**Authors:** Renate Effner, Julia Hiller, Stefanie Eyerich, Claudia Traidl-Hoffmann, Knut Brockow, Massimo Triggiani, Heidrun Behrendt, Carsten B. Schmidt-Weber, Jeroen T. M. Buters

**Affiliations:** 1 Center of Allergy & Environment (ZAUM), Member of the German Center for Lung Research (DZL), Technische Universität München/Helmholtz Center, Munich, Germany; 2 Chair and Institute of Environmental Medicine (UNIKA-T), Technische Universität München and Helmholtz Center Munich, Munich, Germany; 3 CK-CARE, Christine Kühne - Center for Allergy Research and Education, Davos, Switzerland; 4 Department of Dermatology and Allergy, Technische Universität München, Munich, Germany; 5 Division of Allergy and Clinical Immunology, Department of Medicine, University of Salerno, Italy

## Abstract

The mechanisms how environmental compounds influence the human immune system are unknown. The environmentally sensitive transcription factor aryl hydrocarbon receptor (AHR) has immune-modulating functions and responds to small molecules. Cytochrome P4501 enzymes (CYP1) act downstream of the AHR and metabolize small molecules. However, it is currently unknown whether CYP1 activity is relevant for immune modulation. We studied the interdependence of CYP1 and AHR in human primary immune cells using pharmacological methods. CYP1 inhibition increased the expression levels of the stem cell factor receptor (c-Kit) and interleukin (IL)-22 but decreased IL-17. Single cell analyses showed that CYP1 inhibition especially promoted CD4^+^ helper T (Th) cells that co-express c-Kit and IL-22 simultaneously. The addition of an AHR antagonist reversed all these effects. In addition to T cells, we screened other human immune cells for CYP and found cell-specific fingerprints, suggesting that similar mechanisms are present in multiple immune cells. We describe a feedback loop yet unknown in human immune cells where CYP1 inhibition resulted in an altered AHR-dependent immune response. This mechanism relates CYP1-dependent metabolism of environmental small molecules to human immunity.

Environmental pollution influences human immunity creating an increasing burden for health. Key components of pollution are small organic molecules that can interact with the aryl hydrocarbon receptor (AHR), but that are also metabolized by cytochrome P450 (CYP) enzymes. CYP are an ubiquitously expressed, versatile, and conserved enzyme system that metabolizes lipophilic endo- and xenobiotics^1^,^2^. In humans 57 CYP proteins are grouped into 18 families according to their cDNA sequence identities^3^,^4^. Most studied functions of CYPs concern biotransformation reactions with activation of prodrugs or degradation of exogenous substances in the liver. Constitutive extrahepatic expression of CYPs is usually low but can be induced by CYP substrates through ligand-dependent transcription factors such as the AHR^5^.

Upon activation by structurally diverse exogenous or endogenous ligands, the cytosolic AHR translocates into the nucleus and acts as a heterodimeric complex on xenobiotic response elements (XREs)^6^,^7^,^8^,^9^. CYP1 family enzymes, typically regulated by XREs, are markers of AHR activation and could attenuate AHR in a negative feedback pathway^8^,^10^,^11^,^12^,^13^. V*ice versa* inhibition of CYP1 amplified AHR activity in the presence of agonists^14^,^15^. Although AHR was mainly studied in the field of xenobiotic metabolism, this sensor regulates critical immune responses, and thus, translates environmental signals into immunological actions^16^. However, AHR activation by different ligands do not result in one specific immune response but rather in divergent, ligand-dependent immunological outcomes such as inflammation or tolerogenic responses^17^,^18^,^19^. AHR is widely expressed in the hematopoietic system in cells of both innate and adaptive immunity^18^,^20^,^21^,^22^. The pivotal immunological role of AHR is further exemplified by the regulation of the stem cell factor receptor c-Kit, a receptor tyrosine kinase that controls survival and differentiation of immune cells, and by the effects of AHR on the tissue-regulatory cytokines interleukin (IL)-22 and IL-17^23^,^24^,^25^,^26^,^27^. Thus, AHR serves as a relevant factor for epithelial barrier integrity, for autoimmune and atopic diseases and for hematopoietic malignancies^18^,^28^,^29^,^30^,^31^. Although AHR has been intensively studied, to date the role of CYP1 metabolism in human immunity is unclear. We hypothesized that CYP could navigate immune response by degradation of ligands on xeno-sensing transcription factors, and thus may contribute as metabolic keys to immunity. Here, we examined the interdependence of CYP1 and AHR in human immune cells, especially T cells, and analyzed the cell-specific expression of c-Kit and IL-22 during CYP1 inhibition. To test whether similar mechanisms could be active in multiple immune cells, we screened other human immune cell subtypes for constitutive CYP expression. The CYP pathway is engaged in the metabolism of environmental pollutants, drugs and endogenous molecules, and furthermore, previously described enzymatic reactions are known to regulate immune responses^32^,^33^,^34^. Thus the implications of this environmentally triggered feedback pathway may contribute to new options in immune modulation or in tolerance-promoting treatment strategies.

## Results

### CYP1 inhibition induces *KIT* and IL-22 by AHR activation

**Figure 1:**
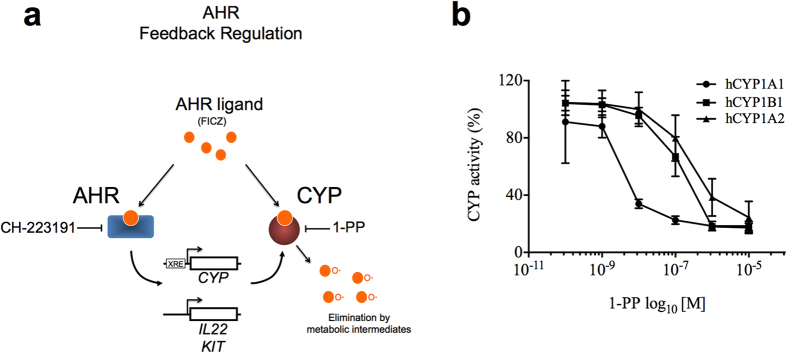
CYP1-dependent AHR activation in human immune cells. (**a**) Graphical Summary. CYP1 enzymes can metabolically inactivate AHR ligands (FICZ) and thereby withdraw these ligands from AHR binding. In the present study, CYP1 suicide inhibitor (1-PP) inhibited degradation of FICZ and increased AHR activity. Consequently, *CYP1*, c-Kit and IL-22 were up-regulated in human immune cells. AHR inhibition by CH-223191 blocked CYP1-induced effects. (**b**) Suicide inhibitor 1-PP inhibited CYP1 activity. Chinese hamster fibroblasts (V79) with stable cDNA-directed expressions of human CYP1 enzymes were pre-incubated with the CYP1A1 suicide inhibitor 1-PP at different concentrations (0.1 nM to 10 μM) for 30 min. 1-PP strongly inhibited human CYP1A1 and to a lesser extent the CYP1A2 and CYP1B1 activities. Catalytic activity was measured by ethoxyresorufin deethylase (EROD) assay. Means ± s.d. of four independent experiments are indicated.

To reduce CYP1 activity (Fig. 1), we used the polycyclic aromatic hydrocarbon (PAH) 1-(1-propynyl)-pyrene (1-PP), which is a selective and efficient mechanism-based (suicide) inhibitor for CYP1A1^35^,^36^. The concentration of 1-PP was optimized in a V79 fibroblast CYP1 expression system with stable cDNA-directed expression of recombinant human CYP1A1, CYP1B1 or CYP1A2 enzymes. 1-PP decreased the activity of human CYP1 assayed as ethoxyresorufin deethylase (EROD) in a concentration-dependent manner (Fig. 1b). CYP1A1 activity was already inhibited by low 1-PP concentrations (IC_50_ = 5 nM), whereas CYP1A2 and CYP1B1 activities were reduced only at higher concentrations (IC_50_ = 650 nM and IC_50_ = 218 nM, respectively). CYP1A1 inhibition was 129-fold and 43-fold more efficient than inhibition of CYP1A2 and CYP1B1, respectively. This selectivity in CYP1 inhibition was not detected with a second general CYP suicide inhibitor 1-aminobenzotriazole (1-ABT) (see Supplementary Fig. S1).

**Figure 2:**
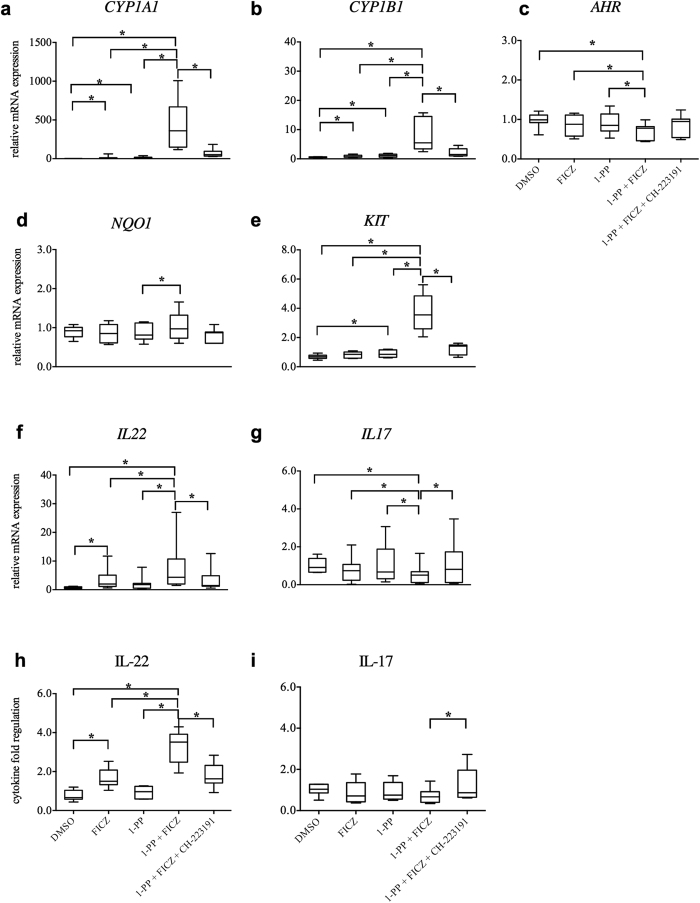
Inhibition of CYP1 activity regulates AHR pathway related targets in human PBMCs. Human PBMCs were activated with anti-CD3/28 antibodies and treated with the AHR agonist FICZ (0.5 nM), the CYP1 inhibitor 1-PP (1 μM) and the AHR antagonist CH-223191 (3 μM) for 5 days. Inhibition of CYP1 activity increased RNA levels of (**a**) *CYP1A1*, (**b**) *CYP1B1,* (**d**) *NQO1*, and the (**e**) *KIT* genes, and slightly down-regulated (**c**) *AHR* transcription levels. CYP1 inhibition also increased IL-22, but decreased IL-17 on RNA (**f** and **g**), and on protein (**h** and **i**) level. Supplementation with the AHR antagonist inverted CYP1-induced effects. RNA expression levels were analyzed by qRT-PCR. Expression values (C_t_ values) were normalized by the housekeeping gene *EF1A* and related to the target gene expression in the medium control (ΔΔC_t_). Data were expressed as 2^−(**ΔΔCt**)^ indicating relative fold regulation. Boxplots show medians, interquartile ranges and ranges of seven different subjects (*p < 0.05 by Wilcoxon signed-rank test).

In order to modulate AHR activity, we used 1-PP together with the high-affinity AHR agonist and CYP1 substrate 6-formylindolo[3,2-*b*]carbazole (FICZ). Appropriate FICZ and 1-PP concentrations for the induction of IL-22 and c-Kit in PBMCs were determined (see Supplementary Fig. S1). We preferred pharmacological inhibition using 1-PP, as molecular genetic transfections influenced T cell viability (data not shown). In order to clarify whether CYP1 inhibition reduces the clearance of FICZ in immune cells, human PBMCs were incubated with combinations of a low dose of FICZ or 1-PP and the AHR antagonist CH-223191 for 5 days (Fig. 2). Known AHR target genes *CYP1A1, CYP1B1,* NAD(P)H-dependent quinone oxidoreductase-1 (*NQO1*), and *KIT* were only marginally up-regulated by FICZ or 1-PP alone but showed significantly (p < 0.05) increased levels when both compounds were combined (compared with FICZ alone: *CYP1A1*: 44.5-fold ± 18.0, *CYP1B1*: 8.6-fold ± 3.6, *NQO1*: 1.2-fold ± 0.2, *KIT*: 4.5-fold ± 0.9; compared with 1-PP alone: *CYP1A1*: 36.0 -fold ± 26.8, *CYP1B1*: 8.3-fold ± 5.0, *NQO1*: 1.2-fold ± 0.2, *KIT*: 4.1-fold ± 0.5, all ± s.d.). Transcription level of *AHR* itself showed a slight but significant down-regulation in co-treated cells, but not in single incubations (compared with FICZ alone: 1.2-fold ± 0.1; compared with 1-PP alone: 1.3-fold ± 0.3, p < 0.05, all ± s.d.) (Fig. 2a–e). The production of IL-22 was significantly induced in co-treated cells at both the RNA (compared with FICZ and 1-PP: 2.4-fold ± 1.0 and 3.6-fold ± 1.3, respectively, p < 0.05, all ± s.d.) and protein level (compared with FICZ and 1-PP alone: 2.0-fold ± 0.4 and 3.8-fold ± 1.2, respectively, p < 0.05, all ± s.d.), whereas IL-17 production was significantly down-regulated (protein: compared with FICZ and 1-PP: 1.1-fold ± 0.2 and 1.3-fold ± 0.3, respectively, p < 0.05; RNA: compared with FICZ and 1-PP: 1.5-fold ± 0.4 and 2.2-fold ± 0.2, respectively, p < 0.05, all ± s.d.) (Fig. 2f–i). After 48 h the induction of IL-22 was concentration-dependent (Supplementary Fig. S2).

Antagonizing AHR by CH-223191 significantly reversed FICZ and 1-PP-induced *CYP1A1* (6.0-fold ± 2.4), *CYP1B1* (3.7-fold ± 0.9)*, KIT* (3.2-fold ± 1.2) and *IL22* (2.6-fold ± 1.4) but restored *IL17* (1.8-fold ± 0.5) expression levels (all ± s.d. and p < 0.05, Fig. 2). The AHR antagonist decreased the effects of an attenuated CYP1 activity almost completely, showing that the target gene regulation was dependent on AHR activation, but also that the AHR agonistic effects of CH-223191 were low. In conclusion, we detected an up-regulation of *CYP1 , KIT* and IL-22 in parallel with a down-regulation of IL-17 through inhibition of CYP1 activity in FICZ-treated human PBMCs. Again, AHR inhibition reversed these effects. The data implicate that a reduced CYP1 activity results in an intracellular accumulation of the AHR ligand FICZ in human immune cells, and that this mechanism regulates immunological AHR targets.

### CYP1 inhibition increases c-Kit and IL-22 in human T cells

**Figure 3:**
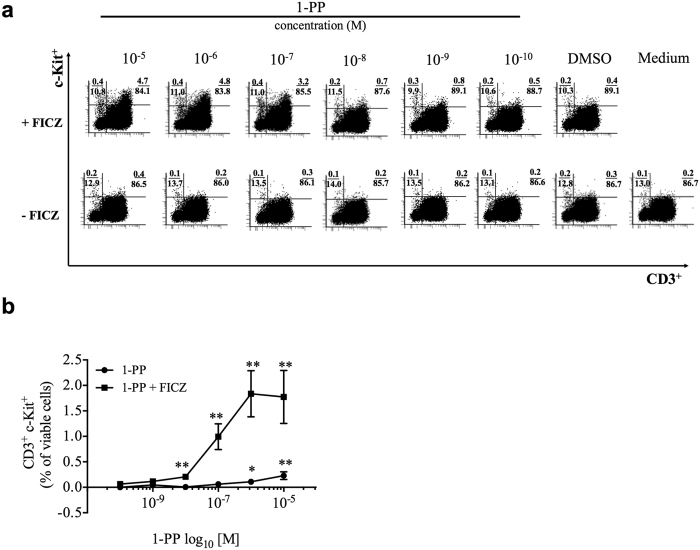
Inhibition of CYP1 activity induces c-Kit on CD3^+^ PBMCs. Human PBMCs were activated with anti-CD3/28 antibodies and treated with increasing concentrations of the CYP1 inhibitor 1-PP (0.1 nM–10 μM) with or without FICZ (0.5 nM), respectively. (**a** and **b**) Concentration-dependent induction of c-Kit on CD3^+^ PBMCs at 48 h with means ± s.e.m. of eight different subjects are shown. *p < 0.05, **p < 0.01 by Wilcoxon signed-rank test.

**Figure 4:**
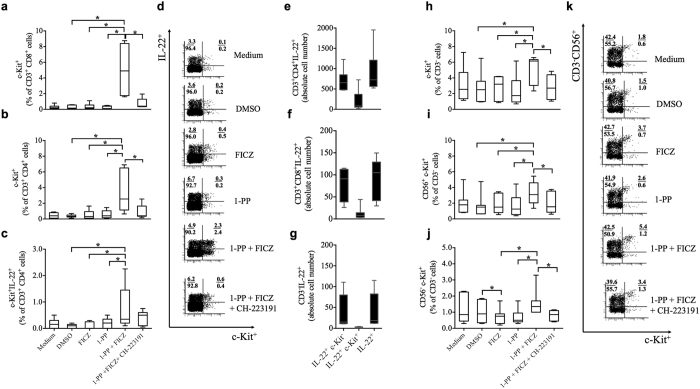
Inhibition of CYP1 activity amplifies c-Kit and IL-22 in human CD3^+^ PBMCs. Human PBMCs were activated with anti-CD3/28 antibodies and treated with the AHR agonist FICZ (0.5 nM), the CYP1 inhibitor 1-PP (1 μM or 0.1 μM for c-Kit^+^/IL-22^+^ PBMCs) and the AHR antagonist CH-223191 (3 μM) for 5 days. Inhibition of CYP1 activity induced c-Kit on (**a**) CD8^+^ Tc cells and on (**b**) CD4^+^ Th cells. (**c** and **d**) The frequency of CD4^+^ Th cells that co-express c-Kit and IL-22 was also augmented in the 1-PP and FICZ co-treatment. (**e–g**) Absolute cell numbers of IL-22^+^ and c-Kit^+^ cells during 1-PP (0.1 μM) and FICZ (0.5 nM) co-treatment. (**h–k**) c-Kit is up-regulated on (**h**) complete CD3^−^ cells, and in detail on (**i–k**) CD3^−^ CD56^+^ and CD3^−^ CD56^−^ PBMCs. All reactions were dependent on AHR activation. Boxplots indicate medians, interquartile ranges and ranges of seven different subjects (*p < 0.05, by Wilcoxon signed-rank test).

We showed at RNA level that treatment by FICZ and 1-PP up-regulated *CYP1A1 , CYP1B1, KIT* and *IL22* in an AHR-dependent manner. We selected c-Kit as an appropriate cell surface receptor to confirm this function of CYP1-induced AHR activation on single immune cells. The addition of the CYP1 inhibitor 1-PP to FICZ substantially increased the expression of c-Kit on CD3^+^ PBMCs in a concentration-dependent manner (Fig. 3a and b). The combined incubation with FICZ, 1-PP and the AHR antagonist CH-223191 was then used to demonstrate the functionality of the AHR feedback loop (Fig. 4). The gating strategies are shown in Supplementary Fig. S3. In the current settings, we measured c-Kit expression on several human immune cell subtypes. In activated PBMCs, CD3^+^ T cells strongly induced the frequency of c-Kit^+^ cells in both CD4^+^ helper T (Th) cells (compared with FICZ alone: 8.8-fold ± 5.2, compared with 1-PP alone: 7.6-fold ± 3.6, p < 0.05, all ± s.d.) and CD8^+^ cytotoxic T (Tc) cells (compared with FICZ: 16.6-fold ± 7.6, compared with 1-PP: 16.5-fold ± 6.7, P < 0.05, Fig. 4a and b, all ± s.d.) revealing that these immune cells are highly sensitive to CYP1-induced AHR activation. Regarding single immune cell analyses of PBMCs we found that the addition of the CYP1 inhibitor to a FICZ-containing medium, at concentrations that showed little effects alone, augmented the simultaneous expression of IL-22 and c-Kit in human CD4^+^ PBMCs. In agreement with the previously shown results, AHR inhibition attenuated the presence of cells that express both IL-22 and c-Kit (Fig. 4c and d).

**Figure 5:**
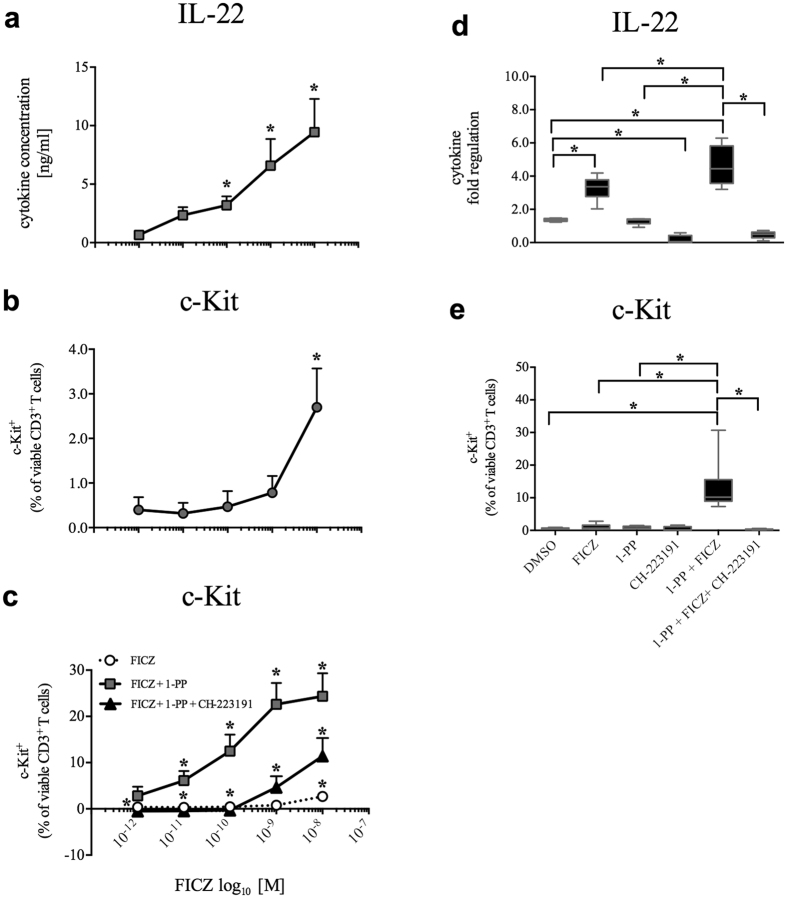
Inhibition of CYP1 activity amplifies c-Kit and IL-22 in purified human CD3^+^ T cells. Human purified and activated CD3^+^ T cells were treated with increasing concentrations of the AHR agonist FICZ (1 pM–10 nM) alone, or in the presence of the CYP1 inhibitor 1-PP (1 μM) and the AHR antagonist CH-223191 (3 μM) for 4 days. (**a** and **b**) Increasing FICZ concentrations induced IL-22 and c-Kit. Data were indicated after subtraction of the DMSO control. (**c**) Induction of c-Kit^+^ T cells. Regulation of CD3^+^c-Kit^+^ T cells by FICZ alone (white circles), by FICZ and 1-PP (grey squares), or by FICZ, 1-PP and CH-223191 (black triangle). Means ± s.e.m. of six different subjects are shown. (**d** and **e**) Regulation of IL-22 and c-Kit in CD3^+^ T cells by FICZ (0.1 nM), 1-PP (1 μM) and CH-223191 (3 μM). Boxplots indicate data of six different subjects with medians, interquartile ranges and ranges (*p < 0.05, by Wilcoxon signed-rank test).

**Figure 6:**
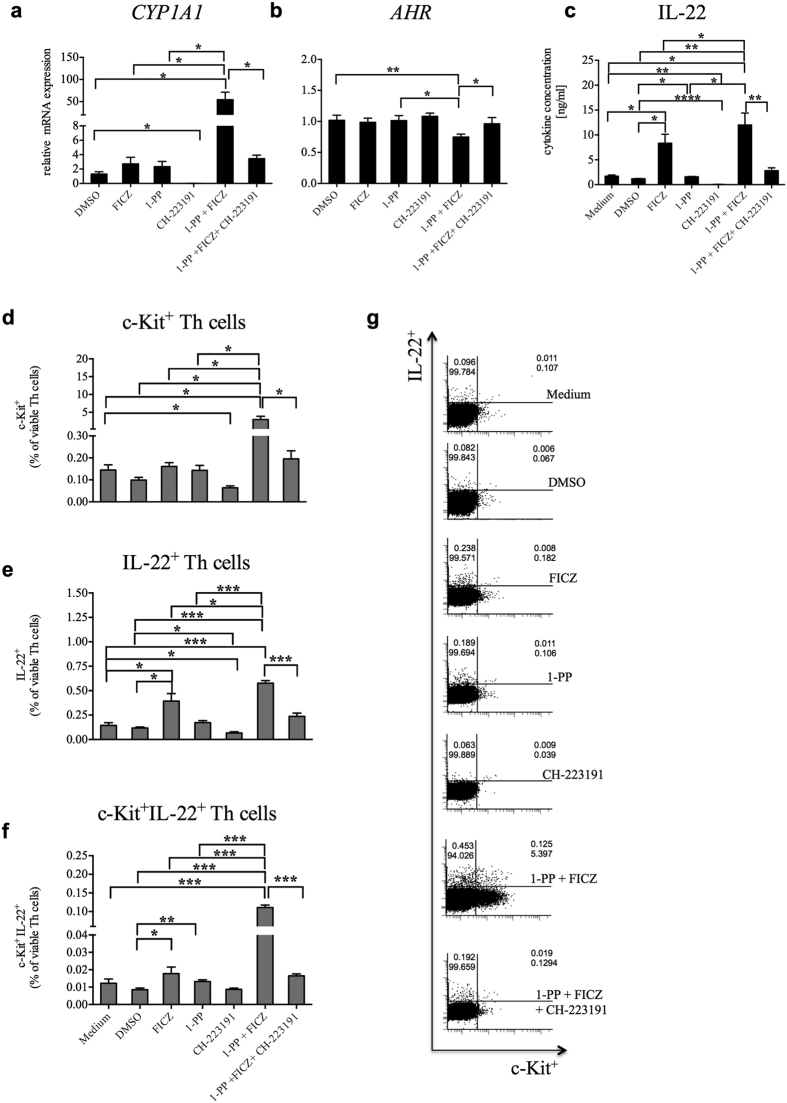
Inhibition of CYP1 activity amplifies *CYP1A1*, c-Kit and IL-22 in purified human CD4^+^ T cells. Human purified and activated CD4^+^ Th cells were treated with the AHR agonist FICZ (0.5 nM) alone or in the presence of the CYP1 inhibitor 1-PP (1 μM) and the AHR antagonist CH-223191 (3 μM) for 4 days. Relative transcription levels of (**a**) *CYP1A1* and (**b**) *AHR* in CD4^+^ Th cells. RNA expression levels were analyzed by qRT-PCR. Expression values (C_t_ values) were normalized by the housekeeping gene *EF1A* and related to the target gene expression in the medium control (ΔΔC_t_). Data were expressed as 2^−(ΔΔCt)^ indicating relative fold regulations. (**c**) Induction of the cytokine IL-22 in CD4^+^ Th cells. (**d–f**) Percentages of CD4^+^c-Kit^+^, CD4^+^IL-22^+^ and CD4^+^c-Kit^+^IL-22^+^ Th cells determined by flow cytometry upon PMA/ionomycin treatment for 5 h. (**g**) Dot plots of treated CD4^+^ Th cells are representative for four different subjects. Shown are means ± s.e.m. (*p < 0.05, **p < 0.01, ***p < 0.001 and ****p < 0.0001 by paired t-test).

Human T cells were highly sensitive to CYP1 inhibition. However, as innate cells express c-Kit more frequently and constitutively than adaptive immune cells^23^,^29^,^37^, we additionally analyzed c-Kit expression on CD3^−^ PBMCs. A reduced CYP1 activity substantially increased c-Kit expression on CD3^−^ cells. c-Kit was significantly up-regulated in potential natural killer cells (CD3^−^CD56^high^) and in CD3^−^CD56^low^ cells (p < 0.05). Innate cells can also produce IL-22, however, as we used CD3/CD28-mediated T cell activation, CD3^−^ cells would be minor contributors to the detected IL-22 levels (Fig. 4e–k, Supplementary Fig. S4). To give more conclusive results, we analyzed AHR activation by CYP1 inhibition in highly purified CD3^+^ and CD3^+^CD4^+^ T cells (Supplementary Fig. S5 and Supplementary Table S1). Purified and activated T cells up-regulated c-Kit and IL-22 with increasing FICZ concentrations (Fig. 5a and b). The CYP1 inhibitor 1-PP strongly amplified FICZ-induced c-Kit and IL-22 (Fig. 5c,d,e). We analyzed *CYP1A1, AHR*, IL-22 and c-Kit regulations in CD4^+^ Th cells. As indicated previously with PBMCs, *CYP1A1* was significantly induced by the combined treatment with 1-PP and FICZ (compared with FICZ and 1-PP alone: 23.5-fold ± 10.9 (s.d.) and 25.3-fold ± 10.1 (s.d.), p < 0.05, respectively), but was significantly down-regulated (15.3-fold ± 5.2 (s.d.), p < 0.05) with the AHR antagonist (Fig. 6a). CYP1 inhibition again decreased the *AHR* transcription (compared with FICZ and 1-PP alone: 1.4-fold ± 0.3 (s.d.) and 1.4-fold ± 0.2 (s.d.), respectively) (Fig. 6b). IL-22 production was significantly up-regulated by FICZ alone (6.9-fold ± 2.2 (s.d.), p < 0.05) and amplified by CYP1 inhibition (compared with FICZ and 1-PP alone: 1.5-fold ± 0.1 (s.d.) and 7.7-fold ± 2.0 (s.d.), respectively, p < 0.05) (Fig. 6c). Again, all these effects were dependent on AHR activation. Single cell analyses showed a substantial induction of c-Kit and/or IL-22-positive Th cells by CYP1 inhibition (Fig. 6d–g, Supplementary Table S2). Majority of c-Kit-positive cells produced IL-22 and not IL-17 (Supplementary Fig. S6). Viability of Th cells during treatment is indicated in Supplementary Fig. S6.

In conclusion single cell analyses of human PBMCs and purified T cell populations indicated that the survival factor and surface receptor c-Kit is a sensitive target for a reduced CYP1 activity, and for AHR activation in various human immune cells, but especially in T cells. An attenuated CYP1 activity promotes human CD4^+^ Th cells that express IL-22 and c-Kit simultaneously. This, to date unknown function of CYP1 enzymes in human primary T cells, confirmed our hypothesis of a CYP1-dependent AHR activation in human immune cells.

### Differential expression of *CYP* and *KIT* in human immune cells

**Figure 7:**
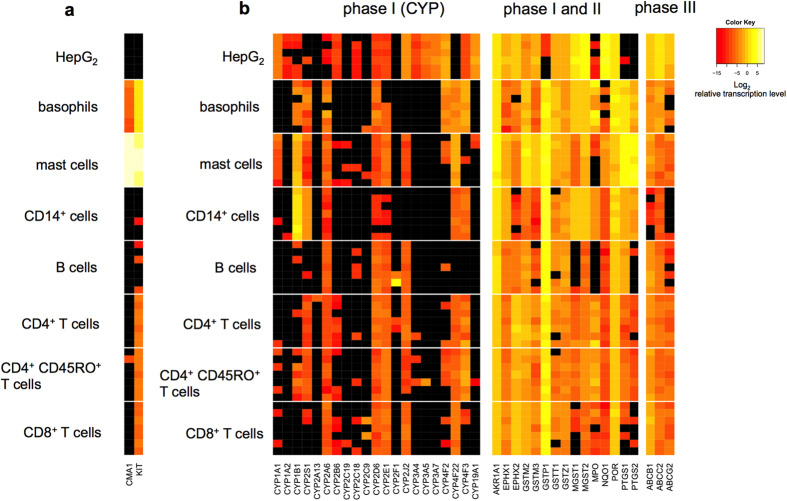
Cell type-specific and constitutive transcription profiles of xenobiotic-metabolizing enzymes in human immune cell subtypes. Human immune cells were isolated from at least seven different donors for each cell type and analyzed without incubation. HepG2 cell line was used as control. Heatmaps show log_2_-transformed relative transcription levels of (**a**) control genes (*CMA1* and *KIT*) or of (**b**) left, CYP1 enzymes (phase I), (**b**) middle, other detoxifying enzymes (phase I and II) and (**b**) right, transporter proteins (phase III). Each row represents one subject; each column represents one gene. Black squares represent not-detected transcripts.

We found an AHR-dependent and CYP1-mediated mechanism in primary T cells, which implies an immune-modulating function of CYP in the human immune system. To further understand the expression pattern of toxicologically important genes in human immune cells, we screened different lymphoid and myeloid immune cells freshly isolated from healthy donors for their RNA level of 39 genes (see Supplementary Table S4) involved in the metabolism of both small environmental and endogenous molecules. The quality control for our screen, the human liver cancer cell line HepG2, widely reflected the expected transcription pattern of xenobiotic-metabolizing enzymes (Fig. 7a and b)^38^. Purities of the cell preparations are shown in Supplementary Table S3. Figure 7 shows the relative transcription levels in different cell types and subjects. We used the *KIT* gene and the mast cell enzyme chymase (*CMA1*) as marker genes for skin-derived human primary mast cells (Fig. 7a). As expected, the *KIT* gene was highly transcribed in human primary foreskin mast cells (122-fold ± 61.2 (s.d.) relative to the *HPRT1* gene). However, the transcription level of *KIT* varied in different immune cell subtypes from healthy subjects. We found a constitutive transcription of the *KIT* gene also in T cells. While *CMA1* was only transcribed in mast cells and basophils, a basal transcription level of the *KIT* gene was detected in basophils (2.4-fold ± 0.4 (s.d.), compared with *HPRT1*), CD4^+^ Th cells (0.02-fold ± 0.01 (s.d.)), CD4^+^CD45RO^+^ memory Th cells (0.04-fold ± 0.01 (s.d.)) and CD8^+^ Tc cells (0.02-fold ± 0.01(s.d.)), but it was barely detected in B cells and CD14^+^ monocytes (Fig. 7a).

Only low transcription levels of different CYP enzymes were detected in human immune cells. CYP1 family members, preferably metabolizing environmental substrates, were differently expressed. Transcription of *CYP1A1* was only detected in primary foreskin mast cells (0.01-fold ± 0.02 (s.d.) compared with *HPRT1*) (Fig. 7b), and in the LAD2 mast cell line (0.07-fold ± 0.02 (s.d.), see Supplementary Fig. S7). *CYP1A2,* which is mainly expressed in liver and lung, was not transcribed in any immune cell. In contrast, several immune cells express *CYP1B1*. We found the highest transcription level in CD14^+^ monocytes (2.5-fold ± 1.0 (s.d.)). *CYP1B1* was also detectable in human primary foreskin mast cells (0.5-fold ± 0.4 (s.d.)) and was constitutively expressed in basophils (0.04-fold ± 0.03 (s.d.)) and in CD4^+^CD45RO^+^ memory Th cells (0.01-fold ± 0.02 (s.d.)). *CYP2D6, CYP2A6* and *CYP2E1* were detected with the highest frequencies among all immune cell subtypes. In contrast to CYP, genes encoding enzymes that are involved in the phase II and phase III detoxification (downstream of CYP, i.e. conjugation-mediated elimination) were expressed ubiquitously. The highest transcription level was found for the gluthation-S-transferase P 1 (GSTP1) gene in all cell populations. In conclusion, each immune cell subpopulation showed a cell type-specific transcription fingerprint of CYP enzymes and besides mast cells and basophils, the c-Kit encoding gene (*KIT*) was constitutively expressed in T cells.

## Discussion

This study demonstrated an environmentally triggered mechanism for immune modulation that is mediated by CYP1 enzymes in humans. These findings are in line with previous reports on a reduced clearance of AHR agonists by CYP1 inhibition that results in a prolonged AHR activation^14^,^15^. The current study depicts a similar mechanism in human peripheral and primary immune cells. CYP1 inhibition increased the AHR targets *CYP1A1* and *CYP1B1* in PBMCs, and antagonizing AHR inverted these effects. Simultaneously, the immunological parameters IL-22 and c-Kit were up-regulated and IL-17 expression was reduced. Induction of c-Kit protein by a reduced CYP1 activity was observed by single cell analyses in a wide range of peripheral human immune cells, especially in CD3^+^, but also in CD3^−^ PBMCs. We found that human CD4^+^ Th cells are highly sensitive to CYP1 inhibition, as they react with the simultaneous expression of c-Kit and IL-22. These results were confirmed with purified T cells. Furthermore, the differential expression of CYP and other drug-metabolizing enzymes in human immune cells implicate cell-specific xenobiotic pathways. In fact, our data imply that numerous human immune cells are prone to AHR feedback activation initiated by xenobiotic-metabolizing enzymes.

The study demonstrated that lymphoid-derived cells especially T cells are susceptible targets for the environmentally induced AHR feedback activation as exemplified by c-Kit induction. c-Kit is important for immune cell differentiation and survival. Immune cells positive for c-Kit are involved in many immunological disorders such as atopic diseases and hematopoietic malignancies^28^,^29^,^30^,^31^.

Inhibition of CYP1 activity during T cell activation and in the presence of a low dose of the AHR agonist FICZ induced IL-22 and c-Kit. Co-expression of c-Kit and IL-22 was especially selected in Th cells. But, only certain subpopulations of Th cells seem to be highly susceptible for the coincident induction of both molecules. These results imply target-specific effects of the AHR agonist FICZ for IL-22 and c-Kit. The expression of AHR in human T cell subsets was previously shown^24^,^39^,^40^. Prigent *et al*. demonstrated an induction of AHR in Th cells during activation and differentiation^39^. The different expression levels of AHR in human Th cell subsets and different activation states of the cells might be crucial for the simultaneous induction of IL-22 and c-Kit. In our experiments, *AHR* transcription decreased with CYP1 inhibition in PBMCs and in Th cells implying that regulation of AHR itself is associated with CYP1 inhibition. We are aware that the frequency of c-Kit^+^IL-22^+^ Th cells is low in the conducted experiments. The c-Kit-ligand and also AHR agonists are present in tissues with close proximity to the environment such as skin^41^,^42^. Therefore, the biological relevance of c-Kit^+^IL-22^+^ Th cells is likely to be dependent on the interaction with tissue cells, where non-hematopoietic cells could retain c-Kit^+^ lymphocytes. Our results indicate that most c-Kit^+^IL-22^+^ Th cells lack an IL-17 production. To our knowledge, however, it is yet unknown whether AHR activation by CYP1 inhibition favors stem cell-like Th22 cells in humans, or whether c-Kit itself regulates IL-22. The here shown, inverse regulation of IL-17 and IL-22 by CYP1-induced AHR activation underlines previous results in humans^24^,^43^ and implies that a reduced CYP1 activity promotes the frequency of human Th22 cells via AHR activation.

IL-22 supports innate immunity by inducing antimicrobial peptides in keratinocytes and regulation of epithelial barrier functions^44^,^45^. Intraepithelial localized Th22 cells are one well-accepted source for IL-22 in humans. While the cytokine is also produced by close to the surface located and c-Kit-positive mast cells, innate lymphoid cells or γδ T cells^29^,^46^, our experiments depicted that CD4^+^ Th cells are the main producers of IL-22.

The expression of AHR in immune cell subtypes is well characterized^18^,^20^,^22^. To our knowledge, however, a comprehensive overview of CYP gene expressions in human uncultured adaptive and innate immune cells was missing. Most studies investigated CYP expression in PBMCs or isolated immune cells after induction or cultivation (summarized in ref. 47). For characterizing constitutive expression levels of xenobiotic-metabolizing enzymes, we used bead-based methods yielding a high cell purity. We avoided culturing of blood cells. Our data showed that immune cell subtypes do not express CYP homogenously. Each immune cell subpopulation is equipped with a unique CYP profile implicating a cell type-specific response to small chemicals. In the present study, the AHR-regulated genes *CYP1A1 , CYP1B1 , NQO1* and the *KIT* gene were all clearly detected in human mast cells, indicating a potential sensitivity to the AHR pathway.

Indeed, AHR is highly expressed in mast cells^48^ and a recent study demonstrated that AHR knockout mice display a reduced number of mast cells and an impaired c-Kit expression^49^. Noteworthy, mast cells were initially defined as the source of IL-22 and dermal mast cells produce IL-22 during infammatory skin diseases^29^,^50^. Regarding the constitutive CYP1 expression in human immune cells from healthy subjects, the AHR-regulated CYP1B1 might be more relevant for environmental substrates than CYP1A1 as shown by the higher frequency and expression level of *CYP1B1* especially in innate leukocytes. This assumption was also reflected by the unexpected result that T cells differ in their *CYP1B1* transcription with a higher transcription level in memory Th cells than in complete Th cells. These data indicate a selective function of the AHR target *CYP1B1* in the immunological memory. Our previous findings of an available c-Kit expression in healthy human T cells was supported by the constitutive *KIT* transcription in T cells including memory Th cells. Although it is unknown to what extent CYP1B1 regulates c-Kit and/or IL-22, the data imply a rapid increase of c-Kit by CYP1 inhibition in primary human T cells that have low constitutive expression levels.

In the present study, we used the endogenously generated AHR agonist FICZ as it is a clearly defined substrate of CYP1 enzymes and a potent AHR agonist^13^,^51^,^52^. We avoided the use of the prototypical AHR agonist and environmental toxin 2,3,7,8-tetrachlorodibenzo-*para*-dioxin (TCDD) in our experiments, as TCDD is slowly metabolized^53^ and amplification of AHR activity by an inhibited metabolism would not occur. We used a low dose of FICZ to induce AHR-mediated effects. CYP1 inhibition augmented FICZ-induced effects most likely by reducing its clearance. However, CYP1 inhibition alone, without additional FICZ, was sufficient for AHR activation in the experiments with PBMCs. This was probably caused by background levels of AHR ligands in the medium resulting from light-induced polymerization of tryptophan^14^,^25^,^54^. Given that FICZ and other AHR ligands are formed endogenously, for example, in the skin^42^, and FICZ metabolites are present in the human urine^55^ we assume that CYP1 in immune cells are induced in a tissue- and cell type-specific manner. CYP1 enzymes are postulated to maintain the steady state of physiological FICZ levels^14^,^22^. Taking into account that various tryptophan derivatives are precursors for FICZ, and its endogenous formation could be regulated by light-dependent and -independent pathways, we do not know to what extent FICZ is physiologically relevant. Competition with other CYP substrates and its rapid metabolism by CYP1 enzymes may regulate its endogenous turnover^56^. We hypothesize that the combination with other AHR ligands and CYP inhibitors elicits similar mechanisms for immune modulation. Furthermore, inter-individual susceptibilities to environmental exposure might be facilitated by genetic variants of CYP enzymes with a reduced or absent catalytic activity^57^. Here, polymorphisms offer a good opportunity to study the physiological CYP function in immunological diseases.

In summary, the present study reveals an active CYP1-dependent AHR activation in human immune cells, especially T cells. We used the surface receptor c-Kit as a marker for CYP1 activity in human T cells. In addition, the study highlights Th cells that simultaneously express IL-22 and c-Kit during AHR feedback activation. Genes encoding CYP enzymes are heterogeneously expressed in primary human immune cells suggesting that similar feedback mechanisms could be present in various cell subtypes. This environmentally triggered and enzymatic mechanism emphasizes a new and significant function of CYP1 enzymes, and reflects a complex and finely regulated interface between the chemical environment and the immune system. The implications of this pathway for sensitization and clinical applications of this mechanism may yield to new options in immune modulation and tolerance-promoting treatment strategies. Indeed tranilast, a non-toxic AHR ligand, is used in several countries as an anti-allergic medication^58^, and we suppose that other AHR ligands or CYP substrates from environmental samples interfering with the xenobiotic-metabolizing system have similar effects.

## Methods

### Subjects

Healthy, non-atopic individuals with total IgE levels <100 kU/l were included in this study. Xenobiotic-metabolizing enzymes were analyzed in at least seven different donors for each cell type. The local ethical committee of the Technical University, Munich, approved the study, and experiments were carried out in accordance with the approved guidelines. Written informed consent from the volunteers involved in this study was obtained.

### CYP1 catalytic activity

V79 Chinese hamster fibroblast cells were grown in a flat-bottom, 96-well plate for 2 days as described previously^59^. 1-(1-Propynyl)-pyrene (1-PP) (synthesized by Prof. A. Seidel, Biochemical Institute for Environmental Carcinogens, Großhansdorf) in DMSO was serially diluted in culture medium to final concentrations of 0.1 nM to 10 μM. CYP activity was measured in V79 cell lines with cDNA-directed expression of human CYP1A1, CYP1A2, and CYP1B1. Cells were pre-incubated with 1-PP, a mechanism-based (suicide) inhibitor for CYP1A1^36^,^60^, for 30 min at 37 °C and rinsed with PBS. Then, 100 μl 1 μM 7-ethoxyresorufin (Sigma-Aldrich, Steinheim, Germany) solved in sodium phosphate buffer (pH 8) was added at 37 °C for 15 min^14^. The reaction was stopped with ice-cold methanol, and the resorufin thus formed was quantified with excitation/emission wavelengths of 544/590 nm. The percentage of inhibition was determined relative to a medium-only control, and the IC_50_ was estimated from the plots.

### Cell culture and stimulation of human peripheral blood mononuclear cells (PBMCs) and T cells

PBMCs from non-atopic, healthy subjects were isolated by density gradient centrifugation and cultured in a flat-bottom 96-well plate coated with 1 μg/ml mouse anti-human CD3 (BD Pharmingen^TM^, Heidelberg, Germany) for 2 h at 37 °C. Cells were cultured in RPMI (Life Technologies, Darmstadt, Germany) supplemented with 5% (v/v) human serum (Lonza, Köln, Germany), 1% (v/v) L-glutamine, 1.12% (v/v) non-essential amino acids, 1.12% (v/v) sodium pyruvate, 1% (v/v) penicillin-streptomycin (Life Technologies), 0.1% (v/v) 50 mM 2-mercaptoethanol and 1 μg/ml mouse anti-human CD28 (BD Pharmingen^TM^). 1-PP was added (0.1 nM–10 μM) in serial dilutions. Then, FICZ (Enzo Life Sciences, Lörrach, Germany) in DMSO was added at a final concentration of 0.5 nM for 48 h. DMSO with and without FICZ served as controls. To adjust the data to the same baseline, we subtracted the control values of DMSO or of FICZ in DMSO from the treatment data. For the 5-day incubations, 1-PP was used with 1 μM and 0.1 μM. DMSO was adjusted to the same concentration in each treatment. The AHR antagonist CH-223191^61^ (Sigma-Aldrich) in DMSO was diluted in cell culture medium to 3 μM. Purified T cells were activated for 4 days under previously described conditions. CD3^+^ T cells were treated with FICZ (1 pM–10 nM) alone, or in combination with the CYP1 inhibitor 1-PP (1 μM) and the AHR antagonist CH-223191 (3 μM). CD4^+^ Th cells were treated as described previously with FICZ (0.5 nM), 1-PP (1 μM) and CH-223191 (3 μM). DMSO was adjusted in all treatments and was below 0.05% in all experiments. Cells and supernatants were harvested after 2, 4 or 5 days for flow cytometry, RNA purification and ELISA. Proliferation was measured by ^3^H-thymidine proliferation assay. The cytokines IL-22 and IL-17 were determined in the supernatants by ELISA (R&D Systems, Wiesbaden, Germany). Cytokine concentrations (pg/ml) were related to proliferation. The medium-only control was used to calculate the fold regulations. Viability was measured by flow cytometry with Fixable Aqua Dead Cell Stain Kit (Life Technologies).

### Flow cytometry

Surface c-Kit expression was analyzed by using mouse anti-human CD117-PE, CD117-APC (A3C6E2) (Miltenyi Biotec, Bergisch-Gladbach, Germany) and mouse anti-human CD3-FITC (OKT3) (eBiosciences, Frankfurt, Germany). After 4 or 5 days of stimulation, PBMCs or T cells were activated with phorbol 12-myristate 13-acetate (PMA) [50 ng/μl] (Sigma-Aldrich) and ionomycin [1 μg/ml] (Sigma-Aldrich) in the presence of monensin (eBiosciences) and GolgiPlug solution (BD Biosciences, Heidelberg, Germany). PMA can influence cell viability and dead cells were excluded by staining with the Fixable Aqua Dead Cell Stain Kit (Life Technologies). For surface receptor staining, mouse anti-human CD3-FITC (OKT3) (eBiosciences), mouse anti-human CD4-APC-Cy7 (RPA-T4) (BD Pharmingen), mouse anti-human CD8-Pacific Blue (RPA-T8) (BD Pharmingen), and anti-human CD56-PE-Vio770 (AF12-7H3) (Miltenyi Biotec) in the presence of FcR Blocking Reagent (Miltenyi Biotec) were used. Cytofix/Cytoperm^TM^ Plus (BD Biosciences) was used for intracellular staining with mouse anti-human IL-22 (Genentech, San Francisco, USA) labeled with Alexa Fluor 647 Microscale Pro (Life Technologies) or mouse anti-human IL-22-PerCP/CY5.5 (2G12A41) (BioLegend, San Francisco, USA) and mouse anti-human IL-17A-PE (eBio64DEC17) (eBioscience). The LSRFortessa^TM^ flow cytometer (Becton Dickinson, Heidelberg, Germany) with appropriate wavelengths was used. Data were analyzed by FACSDiva software (BD Biosciences).

The purity of cells used for the TaqMan Low Density Arrays was determined by flow cytometry as follows: CD14^+^ cells, B cells, and CD4^+^, CD4^+^CD45RO^+^CD45RA^−^ Th and CD8^+^ Tc cells were characterized with mouse anti-human CD14-FITC (61D3) (eBiosciences), mouse anti-human CD45RO-FITC (UCHL1), mouse anti-human CD45RA-PE (HI100), mouse anti-human CD40-FITC (5C3), mouse anti-human CD19-PE (HIB19), mouse anti-human CD8-PE (HIT8a) and mouse anti-human CD4-FITC (RPA-T4) (all obtained from BD Pharmingen), respectively. Human primary foreskin mast cells were analyzed with mouse anti-human CD117-PE (c-Kit, A3C6E2) and basophils with mouse anti-human CD123-PE (AC145) and mouse anti-human CD303-FITC (AC144) (Miltenyi Biotec). Purified CD3^+^ and CD4^+^CD3^+^ T cells were analyzed with mouse anti-human CD3-APC-Cy7 (HIT3a) (BioLegend), and mouse anti-human CD4-V450 (RPA-T4) (BD Biosciences). Purities with representative histograms, and dot plots of isolated T cell populations are indicated in Supplementary Fig. S5 and summarized in Supplementary Table S1.

### Isolation of human primary mast cells, basophils and leukocytes

Mast cells from human foreskins were isolated as described previously^62^. Briefly, dermis and epidermis were separated overnight in 0.05% (w/v) thermolysin type X protease in thermolysin buffer containing 10 mM HEPES, 142 mM NaCl, 6.7 mM KCl, 0.43 mM NaOH, and 1 mM CaCl_2_ at 4 °C. The next day, the dermis was digested in RPMI containing 10% FCS, 0.1% (w/v) collagenase, 0.1% (w/v) hyaluronidase and 0.05% (w/v) DNase (all enzymes obtained from Sigma-Aldrich). Mast cells were purified using CD117 MicroBeads. Blood cells were isolated from at least seven healthy subjects by MicroBead Technology (Miltenyi Biotec). Human primary basophils were purified with the Basophil Isolation Kit II. CD14^+^ cells were isolated using CD14 MicroBeads. Human CD4^+^CD45RO^+^CD45RA^−^ memory Th cells were isolated using the Memory CD4^+^ T Cell Isolation Kit and CD45RA MicroBeads. B cells were purified using the B Cell Isolation Kit II, CD8^+^ Tc cells with the CD8^+^ T Cell Isolation Kit and CD4^+^ Th cells with the CD4^+^ T Cell Isolation Kit II (all from Miltenyi Biotec). CD3^+^ and CD4^+^ T cells were isolated from previously frozen PBMCs by using CD3 or CD4 MicroBeads and the autoMacs Pro Separator (Miltenyi Biotec). To enrich T cells with a high antigen expression by positive selection, beads were used with only ¾ of the recommended volume and T cells were purified twice.

### Cell culture of LAD2 and HepG2 cell lines

The human mast cell line (LAD2) was kindly provided by Prof. Massimo Triggiani and cultured in StemPro-34 medium supplemented with 2.6% (v/v) human serum, 1% L-glutamine (v/v), and 1% penicillin-streptomycin (v/v). Stem cell factor (SCF) (Peprotech, Hamburg, Germany) was added to a final concentration of 100 ng/ml. Cells were cultured at 37 °C with 5% CO_2_. HepG2 cells were cultured in DMEM supplemented with 10% (v/v) fetal calf serum, 2% L-glutamine (v/v), 1% penicillin-streptomycin (v/v) and 1% sodium pyruvate.

### Purification of mRNA, reverse transcription and TaqMan Low Density Arrays

In order to isolate mRNA, RNeasy Kits (Qiagen, Hilden, Germany) with on-column DNase digestion were used. RNA for fingerprinting CYP expression was isolated immediately after cell purifications, and other gene expression analyses were conducted at the indicated time points. The High Capacity cDNA RT Kit (Life Technologies) was used for reverse transcription. Relative transcription levels of *CYP1A1 , CYP1B1 , IL22 , IL17A , KIT, NQO1, AHR* and *EF1A* genes in PBMCs and in CD4^+^ Th cells were measured using FastStart Universal SYBR Green Master (ROX) (Roche, Mannheim, Germany). qPCR primers were obtained from Metabion (Munich, Germany), and target gene transcription levels were normalized to the *EF1A* housekeeping gene. The following primer sequences were used: *AHR*: forward 5′-TCA GTT CTT AGG CTC AGC GTC-3′, reverse 5′-AGT TAT CCT GGC CTC CGT TT-3′; *CYP1A1:* forward 5′-GGA ACC TTC CCT GAT CCT TG-3′, reverse 5′-GGA GAT TGG GAA AAG CAT GA-3′; *CYP1B1:* forward 5′-GCT GCA GTG GCT GCT CCT-3′, reverse 5′-CCC ACG ACC TGA TCC AAT TCT-3′; *IL22*: forward 5′-TCC AGA GGA ATG TGC AAA AG-3′, reverse 5′-ACA GCA AAT CCA GTT CTC CAA-3′; *IL17*: forward 5′-CCA TCC CCA GTT GAT TGG AA-3′, reverse 5′-CTC AGC AGC AGT AGC AGT GAC A-3′; *KIT*: forward 5′-ATG GCA TGC TCC AAT GTG T-3′, reverse 5′-GGC AGT ACA GAA GCA GAG CA-3′; *NOQ1*: forward 5′-ATG TAT GAC AAA GGA CCC TTC C-3′, reverse 5′-TCC CTT GCA GAG AGT ACA TGG-3′; and *EF1A*: forward 5′-CTG AAC CAT CCA GGC CAA AT-3′, reverse 5′-GCC GTG TGG CAA TCC AAT-3′. cDNAs from different human immune cells, the LAD2 mast cell line and HepG2 cells were screened with custom-made microfluidic TaqMan Low Density Arrays (TLDAs) (Life Technologies). The TaqMan assays used can be found in Supplementary Table S4. C_t_ values were normalized to *GAPDH* and depicted relative to *HPRT1* as the ∆∆C_t_ value for each donor and cell type.

### Statistical analysis

Relative transcription levels of genes encoding xenobiotic-metabolizing enzymes in different immune cell subtypes were plotted using the heatmap2 function of the R programming environment^63^. The Wilcoxon signed-rank test for paired samples and the paired t-test were used to test for significant differences. Statistical analysis was performed using GraphPad Prism 6 (San Diego, CA, USA). P-values < 0.05 were considered significant (*); **p < 0.01, ***p < 0.001, ****p < 0.0001.

## Additional Information

**How to cite this article:** Effner, R. *et al*. Cytochrome P450s in human immune cells regulate IL-22 and c-Kit via an AHR feedback loop. *Sci. Rep.* **7**, 44005; doi: 10.1038/srep44005 (2017).

**Publisher's note:** Springer Nature remains neutral with regard to jurisdictional claims in published maps and institutional affiliations.

## Supplementary Material

Supplementary Information
