# Supplementary material for: Cytochrome P450s in human immune cells regulate IL-22 and c-Kit via an AHR feedback loop
Source: Sci Rep. 2017 Mar 9;7:44005. doi: 10.1038/srep44005 (PMC5343665; doi:10.1038/srep44005)
Supplement: Supplementary Information [file srep44005-s1.pdf]

Supplementary Information

Cytochrome P450s in human immune cells regulate IL-22 and c-Kit via an AHR feedback loop

Renate Effner[1], Julia Hiller[2], Stefanie Eyerich[1], Claudia Traidl-Hoffmann[2,3], Knut Brockow[4], Massimo Triggiani[5], Heidrun Behrendt[1], Carsten B Schmidt-Weber[1], Jeroen TM Buters[1,3,*]

[1]Center of Allergy & Environment (ZAUM), Member of the German Center for Lung Research (DZL), Technische Universität München/Helmholtz Center, Munich, Germany

[2]Chair and Institute of Environmental Medicine (UNIKA-T), Technische Universität München, Munich, Germany

[3]CK-CARE, Christine Kühne - Center for Allergy Research and Education, Davos, Switzerland

[4]Department of Dermatology and Allergy, Technische Universität München, Munich, Germany

[5]Division of Allergy and Clinical Immunology, Department of Medicine, University of Salerno, Italy

* Corresponding author

E-mail: buters@tum.de (JTMB)

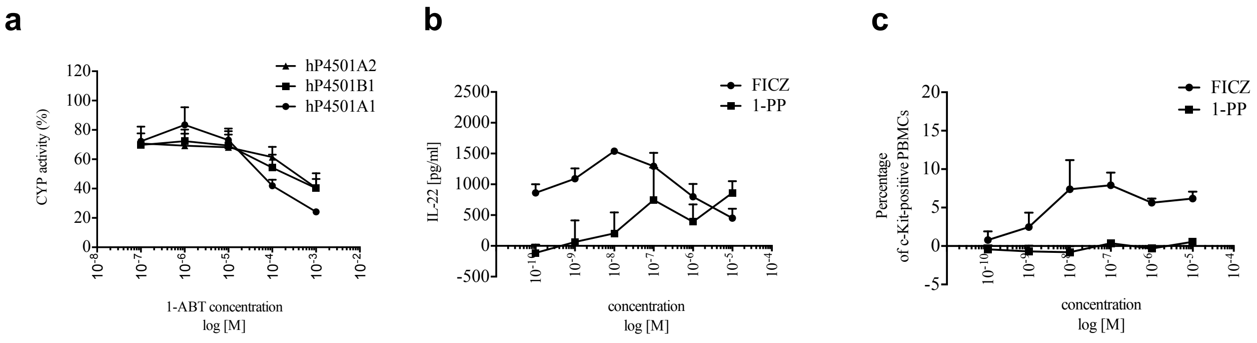

Supplementary Figure S1. Effects of the CYP inhibitor 1-ABT on V79 CYP1 cell lines, and of the CYP1 inhibitor 1-PP and the AHR agonist FICZ on PBMCs. **(a)** Inhibition of CYP1 activity in transfected V79 Chinese hamster cell lines by the CYP suicide inhibitor 1-ABT (10^{-3} M – 10^{-7} M), n=4. **(b)** Regulation of IL-22 (n=3) and **(c)** c-Kit (n=2) by increasing FICZ and 1-PP concentrations of (0.1 nM –10 µM). DMSO control was subtracted from FICZ and 1-PP treatments.

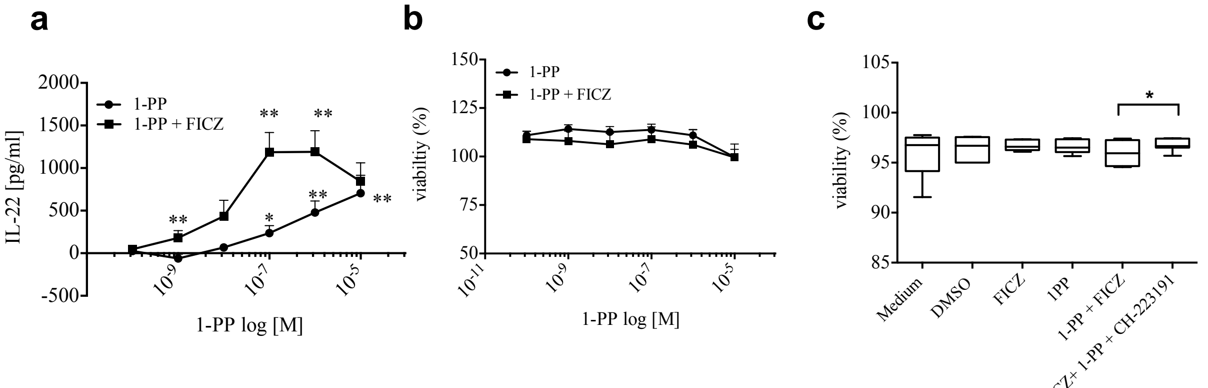

Supplementary Figure S2. IL-22 induction and viability by CYP1 inhibition in human PBMCs. Activated PBMCs were treated with the CYP inhibitor 1-PP (0.1 nM –10 µM) alone or in the presence of the AHR agonist FICZ (0.5 nM). **(a)** IL-22 release and **(b)** viability measured by LDH after 48 h. Means ± s.e.m. of eight independent experiments are shown. *:p<0.05, **:p<0.01. **(c)** Viability of treated PBMCs measured by flow cytometry after 5 days. Boxplots show medians, interquartile ranges (box) and ranges of seven different subjects.

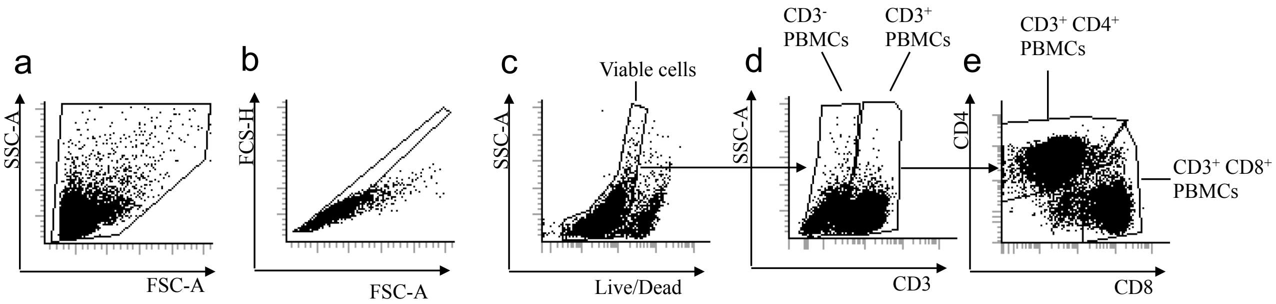

Supplementary Figure S3. Gating strategy of flow cytometry stainings. PBMCs were activated with anti-CD3/CD28 Abs and treated for 5 days. **(a)** PBMCs were collected in FSC-A and SSC-A. **(b-e)** Doublets and dead cells were discriminated and PBMCs were gated for $CD3^{+}CD4^{+}$ and $CD3^{+}CD8^{+}$ cells.

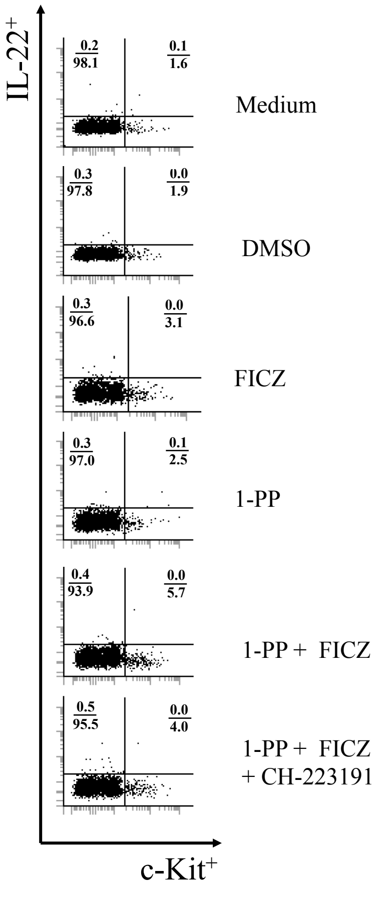

Supplementary Figure S4. Expression of IL-22 in $CD3^{-}$ PBMCs. PBMCs were activated with anti-CD3/CD28 Abs and treated for 5 days. IL-22 expression in $CD3^{-}$ PBMCs was determined by flow cytometry.

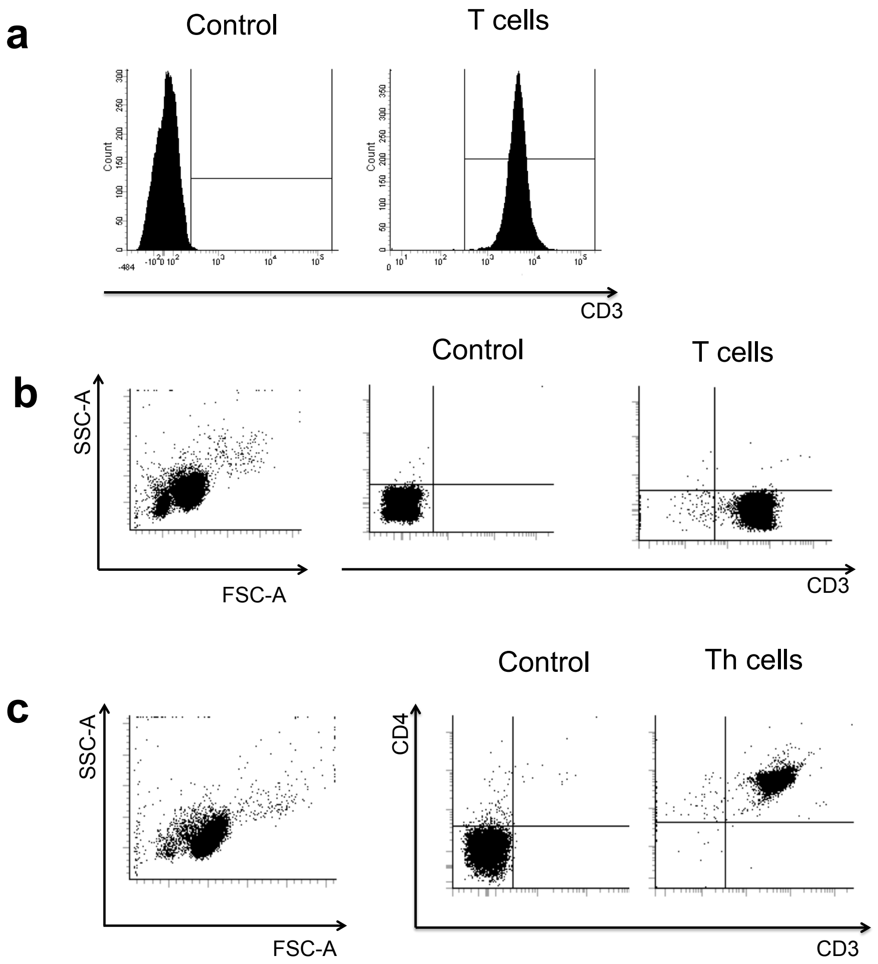

Supplementary Figure S5. Flow cytometry analyses of purified human CD3$^+$ and CD3$^+$CD4$^+$ T cells. T cells were isolated as described in the Method section. **(a** and **b)** Representative histograms and dot plots for CD3$^+$ T cells (CD3 single staining). **(c)** Representative dot plots for CD3$^+$CD4$^+$ Th cells (CD4 and CD3 co-staining).

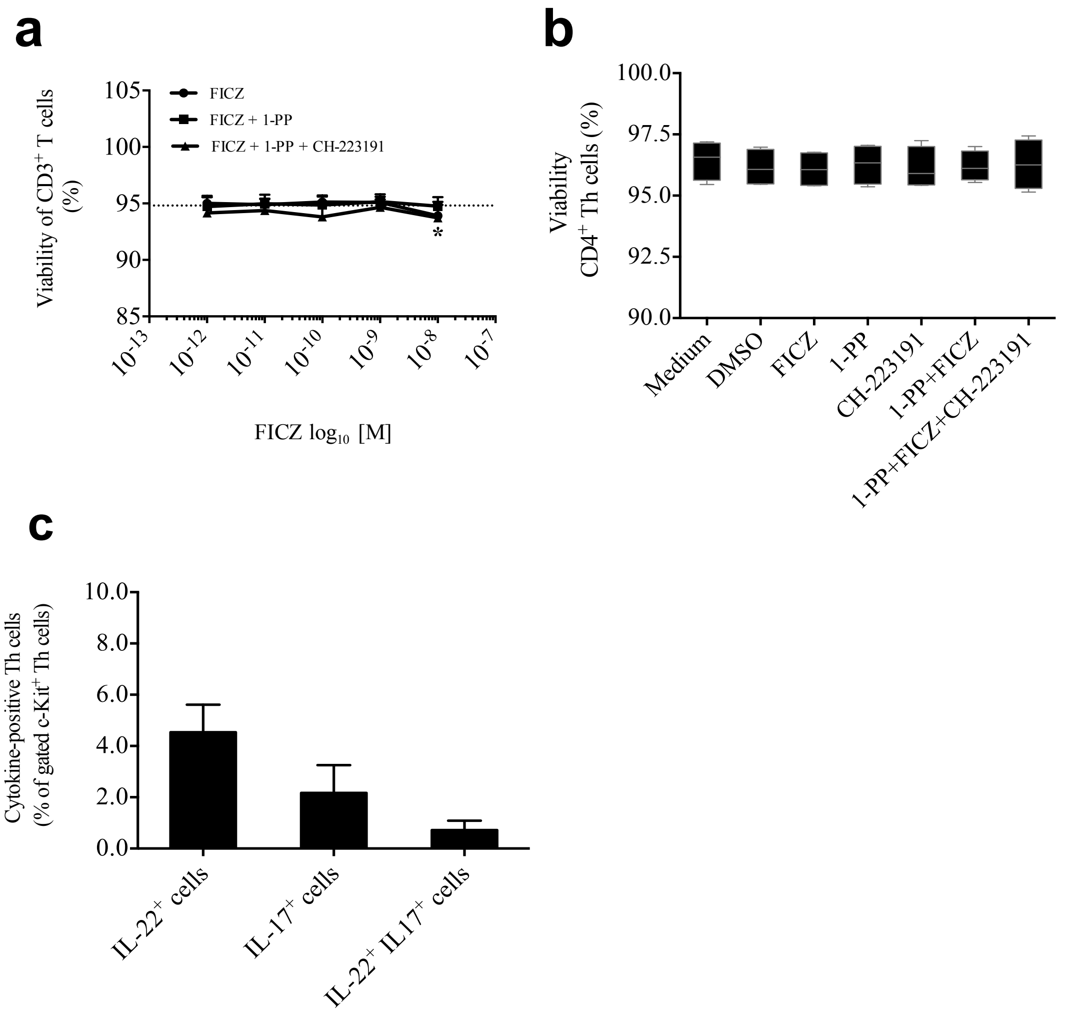

Supplementary Figure S6. Viability of T cells, and IL-22/IL-17 expressions in c-Kit-positive Th cells.

Purified and activated human T cells were treated with the AHR agonist FICZ, the CYP1 inhibitor 1-PP and the AHR antagonist CH-223191 for 4 days. **(a)** Viability of $CD3^+$ T cells after treatment. Means ± s.e.m. of six different subjects are indicated, *$p < 0.05$ by Wilcoxon signed-rank test. Line indicates medium control. **(b)** Viability of $CD4^+$ Th cells after treatment. Viability was determined by flow cytometry. Boxplots show medians, interquartile ranges and ranges of four different subjects. **(c)** Percentages of c-Kit^+IL-22^+, c-Kit^+IL-17^+ and c-Kit^+IL-22^+IL-17^+ Th cells during 1-PP and FICZ treatment determined by flow cytometry. Data are expressed as percentages of gated c-Kit^+ Th cells with means ± s.e.m. of four different subjects.

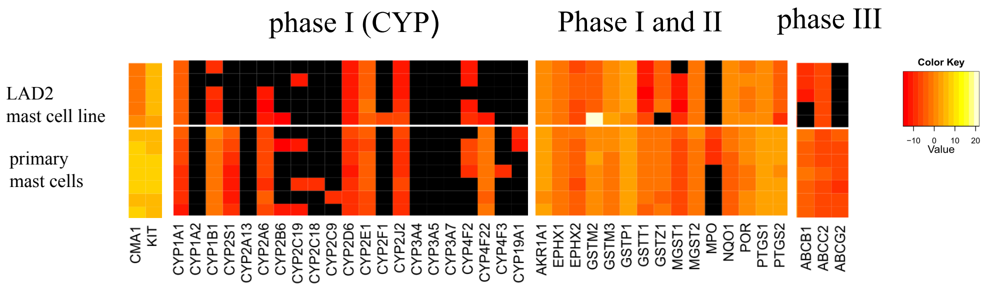

Supplementary Figure S7. Transcription of genes encoding xenobiotic-metabolizing enzymes in the LAD2 mast cell line and in human primary forskin mast cells. Log_2-transformed relative transcription values of *CMA1*, *KIT*, of CYP, phase II and phase III encoding genes are presented as heatmaps. Each row represents one subject, each column a gene. Black squares represent not detected transcripts.

Subject	Purity of CD3$^+$ T cells (%)
1	98.70
2	99.10
3	95.50
4	99.10
5	99.40
6	99.00
Mean	98.47
S.D.	1.34

Subject	Purity of CD3$^+$CD4$^+$ T cells (%)
1	98.90
2	98.40
3	99.20
4	98.90
Mean	98.85
S.D.	0.29

Supplementary Table S1. Purities of human primary CD3$^+$ and CD3$^+$CD4$^+$ T cells. T cells were isolated as described in the Method section and purities were determined by flow cytometry.

abs. cell numbers	c-Kit$^+$	c-Kit$^+$ IL-22$^-$	c-Kit$^+$ IL-22$^+$	c-Kit$^-$ IL-22$^+$	IL-22$^+$
Medium	128.50 ± 14.15	117.50 ± 15.17	11.00 ± 4.08	114.75 ± 20.89	125.75 ± 20.80
DMSO	102.00 ± 27.36	93.25 ± 26.66	8.75 ± 2.06	113.25 ± 23.89	122.00 ± 25.49
FICZ	162.50 ± 36.55	144.50 ± 37.22	18.00 ± 8.04	390.00 ± 145.60	408.00 ± 152.33
1-PP	136.25 ± 31.06	123.50 ± 31.28	12.75 ± 1.70	156.50 ± 47.78	169.25 ± 48.28
CH-223191	64.25 ± 17.28	55.50 ± 16.46	8.75 ± 1.25	58.50 ± 21.97	67.25 ± 22.52
1-PP + FICZ	2908.25 ± 1883.41	2803.25 ± 1870.66	105.00 ± 12.98	440.75 ± 40.30	545.75 ± 44.88
1-PP + FICZ + CH-223191	197.25 ± 77.68	180.75 ± 79.44	16.5 ± 2.08	220.50 ± 62.54	237.00 ± 62.75

Supplementary Table S2. Absolute cell numbers of c-Kit- and IL-22-positive Th cells during CYP1 inhibition.

Th cells were activated and treated with the AHR agonist FICZ (0.5 nM), the CYP1 inhibitor 1-PP (1 µM) and the AHR antagonist CH-223191 for 4 days. Means and standard deviations of four different subjects are indicated.

Cell type	Purity (%)
Basophils	96.1 ± 1.5
Mast cells	95.2 ± 1.8
$CD14^+$ cells	95.8 ± 1.2
B cells	95.8 ± 3.0
$CD8^+$ T cells	93.9 ± 1.7
$CD4^+$ T cells	97.4 ± 0.9
$CD4^+CD45RA^-RO^+$ T cells	92.4 ± 3.7

Supplementary Table S3. Purities of primary cell types used for TaqMan Low Density Arrays. Various immune cell subpopulations were isolated as described in the Method section. Purities of different cell types were analysed by flow cytometry. Means and standard deviations of seven different donors are shown.

Assay ID	Gene Symbol	Gene Name	Chromosome	NCBI Gene Reference
Hs00156558_m1	CMA1	Chymase 1, mast cell	14	NM_001836.2
Hs00174029_m1	KIT	V-kit Hardy-Zuckerman 4 feline sarcoma viral oncogene homolog	4	NM_001093772.1<br>NM_000222.2
Hs00153120_m1	CYP1A1	Cytochrome P450, family 1, subfamily A, polypeptide 1	15	NM_000499.3
Hs00167927_m1	CYP1A2	Cytochrome P450, family 1, subfamily A, polypeptide 2	15	NM_000761.3
Hs00164383_m1	CYP1B1	Cytochrome P450, family 1, subfamily B, polypeptide 1	2	NM_000104.3
Hs00258076_m1	CYP2S1	Cytochrome P450, family 2, subfamily S, polypeptide 1	19	NM_030622.6
Hs00426372_m1	CYP2A13	Cytochrome P450, family 2, subfamily A, polypeptide 13	19	NM_000766.3
Hs00868409_s1	CYP2A6	Cytochrome P450, family 2, subfamily A, polypeptide 6	19	NM_000762.5
Hs00167937_g1	CYP2B6	Cytochrome P450, family 2, subfamily B, polypeptide 6	19	NM_000767.4
Hs00426380_m1	CYP2C19	Cytochrome P450, family 2, subfamily C, polypeptide 19	10	NM_000769.1
Hs00426403_m1	CYP2C18	Cytochrome P450, family 2, subfamily C, polypeptide 18	10	NM_000772.2
Hs00426397_m1	CYP2C9	Cytochrome P450, family 2, subfamily C, polypeptide 9	10	NM_000771.3
Hs00164385_m1	CYP2D6	Cytochrome P450, family 2, subfamily D, polypeptide 6	22	NM_000106.4
Hs00559368_m1	CYP2E1	Cytochrome P450, family 2, subfamily E, polypeptide 1	10	NM_000773.3
Hs00167949_m1	CYP2F1	Cytochrome P450, family 2, subfamily F, polypeptide 1	19	NM_000774.3
Hs00356035_m1	CYP2J2	Cytochrome P450, family 2, subfamily J, polypeptide 2	1	NM_000775.2
Hs00430021_m1	CYP3A4	Cytochrome P450, family 3, subfamily A, polypeptide 4	7	NM_001202855.2<br>NM_017460.5
Hs00241417_m1	CYP3A5	Cytochrome P450, family 3, subfamily A, polypeptide 5	7	NM_001190484.1
Hs00426361_m1	CYP3A7	Cytochrome P450, family 3, subfamily A, polypeptide 7	7	NM_000765.3
Hs00426608_m1	CYP4F2	Cytochrome P450, family 4, subfamily F, polypeptide 2	19	NM_001082.3
Hs00403446_m1	CYP4F22	Cytochrome P450, family 4, subfamily F, polypeptide 22	19	NM_173483.3
Hs00168521_m1	CYP4F3	Cytochrome P450, family 4, subfamily F, polypeptide 3	19	NM_000896.2
Hs00240671_m1	CYP19A1	Cytochrome P450, family 19, subfamily A, polypeptide 1	15	NM_031226.2<br>NM_000103.3
Hs00195992_m1	AKR1A1	Aldo-keto reductase family 1, member A1 (aldehyde reductase)	1	NM_153326.2<br>NM_001202413.1<br>NM_001202414.1<br>NM_006066.3
Hs00164458_m1	EPHX1	Epoxide hydrolase 1, microsomal (xenobiotic)	1	NM_001136018.2<br>NM_000120.3
Hs00157403_m1	EPHX2	Epoxide hydrolase 2, cytoplasmic	8	NM_001979.4
Hs00265266_g1	GSTM2	Glutathione S-transferase mu 2 (muscle)	1	NM_01142368.1<br>NM_000848.3
Hs00356079_m1	GSTM3	Glutathione S-transferase mu 3 (brain)	1	NM_000849.4
Hs02512067_s1	GSTP1	Glutathione S-transferase pi 1	11	NM_000852.3
Hs00184475_m1	GSTT1	Glutathione S-transferase theta 1	22	NM_000853.2
Hs00155313_m1	GSTZ1	Glutathione transferase zeta 1	14	NM_145870.2<br>NM_145871.2
Hs00220393_m1	MGST1	Microsomal glutathione S-transferase 1	12	NM_145791.1

				NM_145764.1<br>NM_145792.1<br>NM_020300.3
Hs00182064_m1	MGST2	Microsomal glutathione S-transferase 2	4	NM_001204366.1<br>NM_002413.4
Hs00165162_m1	MPO	Myeloperoxidase	17	NM_000250.1
Hs00168547_m1	NQO1	NAD(P)H dehydrogenase, quinone 1	16	NM_001025434.1<br>NM_000903.2<br>NM_001025433.1
Hs00287016_m1	POR	P450 (cytochrome) oxidoreductase	7	NM_000941.2
Hs00924803_m1	PTGS1	Prostaglandin-endoperoxide synthase 1 (prostaglandin G/H synthase and cyclooxygenase)	9	NM_080591.1<br>NM_000962.2
Hs00153133_m1	PTGS2	Prostaglandin-endoperoxide synthase 2 (prostaglandin G/H synthase and cyclooxygenase)	1	NM_000963.2
Hs00184500_m1	ABCB1	ATP-binding cassette, sub-family B (MDR/TAP), member 1	7	NM_000927.4
Hs00166123_m1	ABCC2	ATP-binding cassette, sub-family C (CFTR/MRP), member 2	10	NM_000392.3
Hs00184979_m1	ABCG2	ATP-binding cassette, sub-family G (WHITE), member 2	4	NM_004827.2
Hs99999905_m1	GAPDH	Glyceraldehyde-3-phosphate dehydrogenase	12	NM_002046.3
Hs99999909_m1	HPRT1	Hypoxanthine phosphoribosyltransferase 1	X	NM_000194.2

Supplementary Table S4. Selected Assay IDs for TaqMan Low Density (TLDA) Arrays.
